# Metabolomics in systemic sclerosis

**DOI:** 10.1007/s00296-024-05628-y

**Published:** 2024-07-09

**Authors:** Zuzanna Gogulska, Zaneta Smolenska, Jacek Turyn, Zbigniew Zdrojewski, Michał Chmielewski

**Affiliations:** 1 Department of Rheumatology, Clinical Immunology, Geriatrics and Internal Medicine, Medical University of Gdansk, Gdansk, Poland; 2 Department of Biochemistry, Medical University of Gdansk, Gdansk, Poland

**Keywords:** Systemic sclerosis, Metabolomics, Metabolome

## Abstract

Systemic sclerosis is a rare autoimmune condition leading to incurable complications. Therefore fast and precise diagnosis is crucial to prevent patient death and to maintain quality of life. Unfortunately, currently known biomarkers do not meet this need. To address this problem researchers use diverse approaches to elucidate the underlying aberrations. One of the methods applied is metabolomics. This modern technique enables a comprehensive assessment of multiple compound concentrations simultaneously. As it has been gaining popularity, we found it necessary to summarize metabolomic studies presented so far in a narrative review. We found 11 appropriate articles. All of the researchers found significant differences between patients and control groups, whereas the reported findings were highly inconsistent. Additionally, we have found the investigated groups in most studies were scarcely described, and the inclusion/exclusion approach was diverse. Therefore, further study with meticulous patient assessment is necessary.

## Introduction

Systemic sclerosis (SSc) is a rare disease with a prevalence of 7.2–33.9 per 100,000 in Europe [1], although its incidence keeps increasing. Its pathology is complex. Vasculopathy, immune system disturbances, and fibrosis, presumably triggered by environmental factors, are the leading aberrations [2]. Moreover, growing data impute that diet and lifestyle affect the development and course of rheumatological conditions, thus they cannot be neglected [3].

The SSc hallmark features are skin thickening and the Raynaud phenomenon (RP). Notwithstanding, organ involvement is a common complication that implicates diverse prognoses. The risk may be clinically stratified by assignment into a limited SSc (lcSSc) type or a diffuse SSc (dcSSc) type; by assessment of risk factors like patients’ sex or age at onset, and by serological testing for specific autoantibodies. Nevertheless, the current knowledge often fails to predict particular organ involvement and overall prognosis. Thus, specific biomarkers discovery is needed.

In this light, metabolomics emerges as a potential tool to elucidate those processes and may lead to new biomarkers discovery. This modern approach provides a comprehensive study of human metabolism, by detection, concentration measurement (and its simultaneous comparison) of molecules smaller than 1 kD [4]. This hypothesis-free approach uses advanced biochemical techniques like liquid chromatography-mass spectrometry (LC-MS), gas chromatography-mass spectrometry (GC-MS), or nuclear magnetic resonance (NMR) spectroscopy to create a “metabolomic fingerprint” of studied samples [5]. NMR spectroscopy interprets radiation of electromagnetic waves of excited atom nuclei. The running assay cost is comparably lower in comparison to chromatography-mass spectrometry, and the compounds are not destroyed during the analysis, therefore they can serve for further tests. Sample preparation is quick, and this technique allows detailed detection and quantification of isomers. The minimal detection range is nanomolar, which is considered high in comparison to other techniques [4]. The GC-MS and LC-MS technique uses ionization and consecutively ion acceleration through electromagnetic fields. Detection and analysis of their path lead to the mass-to-charge (m/z) ratio measurement, which enables substance identification. Though a minimal sample is required, its preparation is complicated and the sample cannot be recovered [4]. The major difference between LC-MS and GC-MS is the mobile phase used (liquid and gas respectively). After detection, the identification can be made using databases like The Human Metabolome Database [6], which currently lists more than 41,000 metabolites.

In the last decade, we observed the data on SSc metabolism was growing. Therefore we found it necessary to summarize the research performed so far, point to conclusions that resulted from those studies, and highlight the problems that still are not addressed properly.

## The aim of the narrative review

Our paper aims to highlight the need for further research on SSc and its biomarkers, especially using metabolomic methods. Therefore, we summarize current knowledge about serum or plasma metabolite aberrations in SSc patients.

## Search strategy

**Fig. 1:**
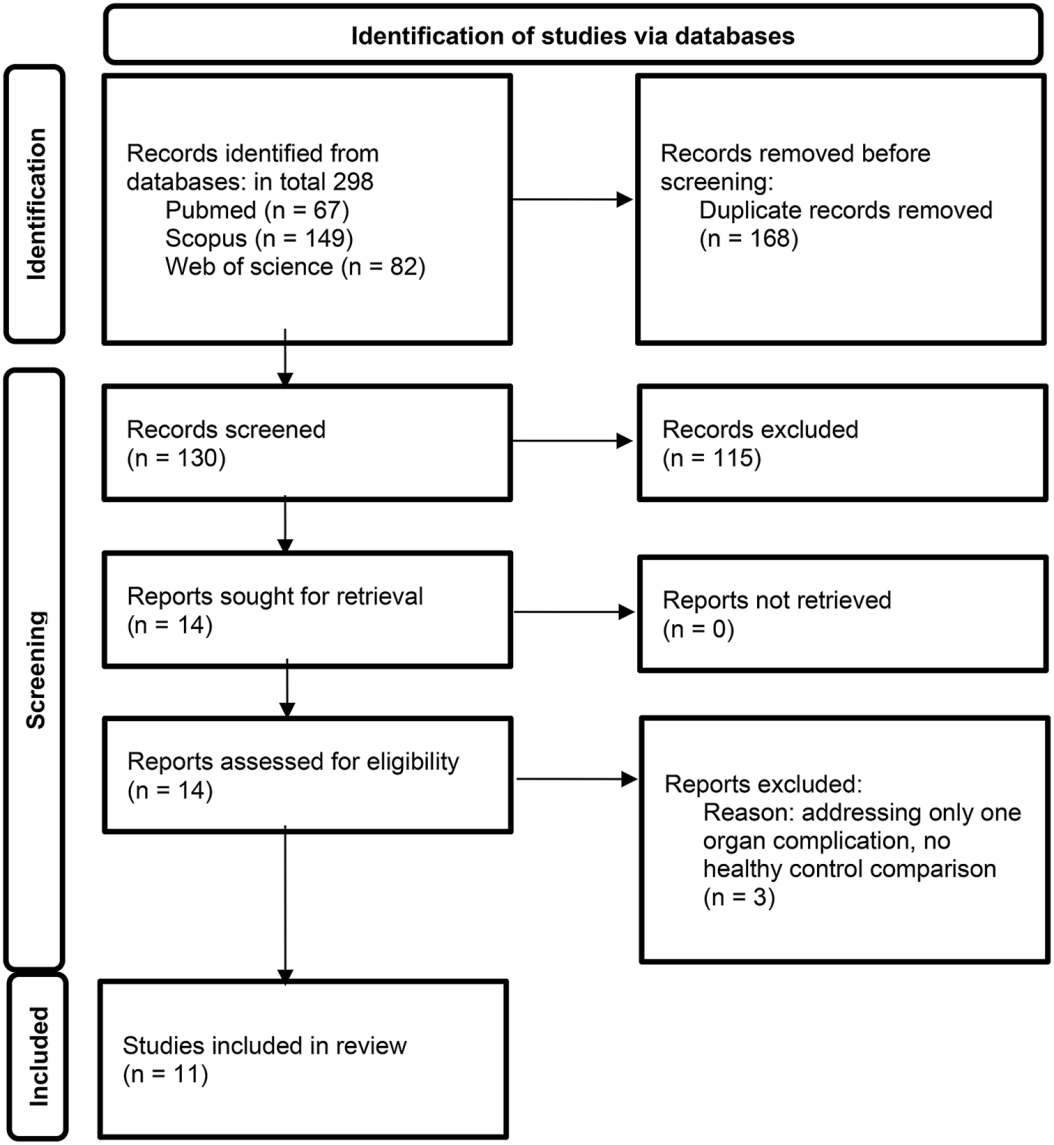
Prisma 2020 flow diagram [41]

On 21st March 2024, a narrative review of all-time published papers using PubMed, Scopus database, and Web of Science Database was performed. Questions inserted were: (metabolomics) AND (“systemic sclerosis”), (metabolomics) AND (scleroderma), (chromatography-mass spectrometry) AND (“systemic sclerosis”), (chromatography-mass spectrometry) AND (scleroderma), (“NMR”) AND (“systemic sclerosis”), (“NMR”) AND (scleroderma) in all fields for PubMed, and Web of Science databases, in title, abstract, and keywords for Scopus database. We applied the English language as a filter, then excluded duplicates, and read the remaining abstracts. Only original papers focusing on serum or plasma metabolome in SSc were included (Fig. 1.).

## Results

298 papers in total were found. After elimination according to methods presented above we found 11 papers that fully met our criteria. Table 1. presents their summary, including characteristics of the investigated groups, applied techniques, and the aim of the papers. Additionally, in a separate chapter, we discuss 3 papers published so far, that addressed metabolomics of pulmonary arterial hypertension (PAH) in SSc. This specific complication was in the spotlight of the researchers, because of its promising implications. PAH is one of the leading causes of death among SSc patients. Lately, we observed breakthroughs in SSc-PAH treatment prolonging the patients’ lifespan. The drugs introduced into therapy were discovered after thorough biochemical studies on impaired endothelial metabolism in this group of patients. Because of the narrow focus of quoted works, those findings cannot be compared to other metabolomic studies on SSc.

**Table 1:** Papers included in the review

	SSc patients	Methods applied	Specific remarks
Bellocchi et al. (2018) [12]	59 (88,1% female, 17,1% dcSSc,39% ACA-positive, 39% anti-scl70 positive)	LC-MS, plasma analysis	special attention to intestinal involvement and microbiota disturbances
Bengtsson et al. (2016) [8]	19 (84% female, 52,6% dcSSc, 42,1% ACA positive, 21%, anti-Scl70 positive)	GC-MS, serum analysis	SSc is included as a control to compare to SLE,
Bögl et al. (2022) [13]	52 (84,6% female, 21,2% dcSSc, 34,6% ACA positive, 32,7 anti-Scl70 positive)	LC-MS, plasma analysis	SSc vs. HC
Fernandez-Ochoa et al. (2019) [15]	59 (88,1% female, 16,9% dcSSc, 39% ACA positive, 39% anti-Scl70 positive)	LC-MS, plasma and urine analysis	SSc vs. HC
Fernandez-Ochoa et al. (2020) [7]	43 (83,6% female)	LC-MS, plasma and urine analysis	focused on connective tissue diseases (RA, SSc, SLE, SjS, UCTD, MCTD, PAPS) vs. healthy controls
Guo et al. (2023) [9]	127 non-treated (59,8% female, 57 treated (59,6% female)	LC-MS, serum analysis	human and mouse models, treated and non-treated before patients
Meier et al. (2020) [10]	36 (83,3% female, 25% dSSc, 33,3% ACA positive, 27,8% anti-Scl70 positive)	LC-MS, serum analysis	SSc vs. HC and non-ILD-SSc vs. stable ILD-SSc vs. progressive ILD-SSc
Murgia et al. (2018) [16]	37 (78,4% female, 38% dcSSc, 31% ACA positive, 34% anti-Scl70 positive)	GC-MS, NMR, serum analysis	SSc vs. HC
Ottria et al. (2020) [14]	discovery cohort 20 (85% female, 35% dcSSc, 55% ACA positive, 25% anti-scl70 positive) validation cohort 12 (92% female, 16,7% dcSSc, 25% ACA positive, 50% anti-scl70 positive)	discovery group LC-MS validation group GC-MS (carnitine), fatty acid analysis (LC-MS), plasma	SSc vs. HC
Smolenska et al. (2020) [17]	42 (83,3% female, 50% dcSSc)	LC-MS plasma analysis	focused on aminoacids and their derivatives
Sun et al. (2023) [11]	30 (80% female, 40% dcSSc,	LC-MS, serum analysis	SSc vs. HC

*ACA (anti-centromere antibodies), dcSSc (diffuse systemic sclerosis), GC-MS (gas chromatography-mass spectrometry), HC (healthy controls), LC-MS (liquid chromatography-mass spectrometry), MCTD (mixed connective tissue disease), NMR (nuclear magnetic resonance), RA (rheumatoid arthritis), SjS (Sjogren’s syndrome), SLE (systemic lupus erythematosus), SSc (systemic sclerosis), UCTD (undifferentiated connective tissue disease*)

### Holistic studies on systemic sclerosis metabolome disturbances

We found 11 papers addressing this problem, 2 of them used SSc as a control group while focusing on other rheumatological conditions [7, 8]. The number of SSc patients included in particular studies varied from 19 to 127. In all studies, women constituted the majority of patients. Most researchers reported the serological status and clinical presentation (lcSSc/dcSSc) of patients in the studied group. 6 studies used plasma and 5 studies used serum in the analysis. All papers found significant differences between SSc and healthy controls (HCs) metabolome, but only 7 investigated a correlation between clinical subtype or organ involvement.

The work of Guo et al. [9] should be highlighted explicitly as they conducted the most comprehensive research that analyzed separately newly diagnosed patients (without previous treatment), those who already started therapy, HCs, and additionally mouse models of the disease. They proved that certain patterns could clearly distinguish patients without previous treatment from HCs. They found 846 substances in significantly different concentrations. They chose 164 of them for further analysis (KEGG pathway enrichment analysis) and proved that untreated SSc patients’ sera presented enhanced amino-acid and pyruvate metabolism as well as the upregulation of pentose phosphate pathway, pathways related to glycolysis, bile acid biosynthesis (increase: taurine, hypotaurine, cysteine, and lysine related metabolites). It also showed decreased α-linolenic, linolenic acid, and fatty acids metabolism. The researchers also proved that treated SSc patients could be separated from untreated patients, and HCs regarding their metabolome profile. They found 506 metabolite aberrations in univariate analysis. They selected 212 of them for further study of KEGG pathway enrichment analysis. These upregulated processes included glycometabolism (lactose degradation), amino-acid metabolism (phenylalanine, tyrosine, glycine, serine, and betaine metabolism), urea cycle, and pyruvaldehyde degradation. Downregulated processes were α-linolenic acid, linoleic acid, biotin metabolism, citric acid cycle, as well as certain amino-acid metabolism (methylhistidine, homocysteine), phosphatidylcholine biosynthesis, ubiquinone biosynthesis. They presented that amidosulfonic acid, all-trans-retinoic acid, L-proline, L-glutamic acid, betaine, uracil, phthalic acid, guanidinosuccinic acid, and isovalerylglycine levels tend to normalize after treatment. Moreover, the researchers analyzed changes in the metabolomic profile depending on skin changes regression. They found some of the aberrated metabolites (γ-carboxyethyl hydroxychroman (γ-CEHC), paraxanthine, PS(18:0/18:1(9z)), 2,3-diaminosalicyclic acid, MG(0:0/182(9Z,12Z)/0:0), and phloretin 2’-O-glucuronide) being normalized following treatment in the skin-improvement SSc group. Moreover, they found distinctive metabolites for certain clinical presentations. Among the compounds negatively correlated with skin involvement (measured with modified Rodnan skin score, mRSS) they found allysine and all-trans-retinoic acid (also correlating with inflammatory parameters). The markers that positively correlated with mRSS were: of D-glucuronic acid, hexanyolcarnitine. Biomarkers for skin progression were: mediagenic acid 3-O-β-D-glucuronide, 4’-O-methyl-(-)-epicatechin-3’-O-β-glucuronide, valproic acid glucuronide. Sclerodactyly was negatively associated with thromboxane B_2_ (whereas there was a positive correlation with abnormal capillaroscopy findings); and positively with phthalic acid. They described that L-tryptophan negatively correlates with laboratory inflammatory markers. Moreover, ILD diagnosis correlated with γ-linolenic acid, dihydrothymine, etiocholanolone glucuronide, L-pipecolic acid, carnosine, and L-cystathione (positively); proline, betaine, androsterone sulfate, phloretin 2’-O-glucuronide, 4-guanidinobutanoic acid, and NNAL-N-glucuronide (4-(methylnitrosamino)-1-(3-piridyl)-1-butanol N-glucuronide (negatively).

Those results are inconsistent with other reports. Meier et al. [10] who paid special attention to ILD development and progression used targeted LC-MS (110 molecules), to discover 85 distinctive substances. They reported that levels of L-threonine, xanthosine, 3-aminoisobutyric acid, leucine, isoleucine, and adenosine monophosphate correlated with ILD presence and corresponded with deterioration in lung function tests. Additionally, they proposed the SSc-biomarkers (L-thyrosine, leucine, isoleucine, and L-tryptophan) and created a metabolomic distinguishing model. Other SSc-ILD biomarkers (L-glutamine and Ile-Ala) were proposed by Sun et al. [11] who performed untargeted research and found 38 compounds of different concentrations, 32 of which presented good diagnostic value (including vitamin E, various lipid metabolites, and amino acids). Moreover, they observed distinctive patterns for dcSSc and lcSSc subtypes.

On the other hand, next authors [12] detected no metabolomic difference between patients with or without ILD. In this paper, a model of 17 metabolites that allowed SSc differentiation from HCs was proposed. Additionally, researchers found 7 single metabolites that distinguished SSc patients from HCs (diacylglycerol 38:5, phosphatidylcholine 36:4, 1-(9Z-pentadecenoyl)-glycero-3-phosphate, DL-2-aminooctanoic acid, 2,4-dinitrobenzenosulfonic acid, α-N-phenylacetyl-L-glutamine, one was unidentified). The authors found that intestinal microbiota examination might distinguish SSc patients from HC (reduced prevalence of 9 bacterial species: from Firmicutes, Bacteroidetes, and Proteobacteria family). This disturbance may lead to the lower production of butyrate, which importance is outlined in the following discussion.

Metabolomic proof for gut microbiome dysregulation was also reported by Bögl et al. [13]. They performed both targeted and untargeted assays and recognized 29 aberrated metabolites from different biochemical groups, mainly amino acids (dimethylarginine, citrulline, ornithine, 1-methylhistidine, taurine, 3-methylhistidine, tryptophan, alanine, tyrosine, methionine, lysine, proline), their derivatives and other (kynurenine, TMAO, trimethyllysine, hexanoylcarnitine, acetylcarnitine, choline, octanoylcarnitine, valerylcarnityne). Untargeted analysis found additional aberrations in lysophosphatidylcholine (LPC) 22:4 and 22:2, sphingomyelins (SM) 32:2, and 34:1, OH-tryptophan, phenylacetylglutamine (PAG), OH-butyrylcarnitine, OH-decanoylcarnitine), and multiple other unrecognized molecules. They performed a complex analysis of the aberrations and proposed metabolomic pathways that were imbalanced in SSc patients: kynurenine pathway, urea cycle, lipid metabolism, and a disturbed gut microbiome.

Further proof for pathological lipid metabolism in SSc was reported by Ottria et al. [14]. The authors highlighted that in their study L-carnitine and acylcarnitines were the most relevant biomarkers of SSc. Via an untargeted LC-MS analysis, 56 aberrated metabolites were detected, which suggested disturbed fatty acid oxidation, and impaired kidney function in the SSc group. Furthermore, the authors performed a targeted analysis focusing on fatty acids and carnitine concentrations. They confirmed the suggested disturbances and noted differences in lauric acid, myristic acid, arachidic acid, carnitine, isovalerylcarnitine, octanoylcarnitine, palmitoylcarnitine concentrations. Additionally, they reported that adding carnitine transporter inhibitors to healthy and SSc dendritic cells inhibited pro-inflammatory response.

Another approach was examining both plasma and urine samples. Some reports [15] present the detection of plasma 46 metabolites with concentrations different than in HCs, unfortunately only 7 were recognized (butyrylcarnitine, valerylcarnitine, α-N-phenylacetyl-L-glutamine, 2-4-dinitrobenzenesulfonic acid, MG (20:5), 1-arachidonoylglycerol, oleic acid). 12 plasma metabolites were used to create a differentiating model. The authors additionally compared lcSSc and dcSSc groups and interstitial lung disease (ILD) vs. non-ILD patients and found no significant differences among plasma metabolites. On the other hand, they found that some urine metabolites could become biomarkers for SSc-associated ILD (SSc-ILD). Additionally, the authors highlighted 2-arachidonoylglycerol as a possible biomarker of SSc. This metabolite plays a role in the endocannabinoid system and may be involved in autoimmunity. In a later study [7] the researchers again present a 12-metabolite classifying model to differentiate SSc from other connective tissue diseases. Unfortunately, the authors did not provide inform ation on specific aberrations found in SSc, as they focused on differentiating mixed connective tissue disease (MCTD) and undifferentiated connective tissue disease (UCTD). Interestingly they found stronger accuracy for urine metabolites as biomarkers, especially in rheumatoid arthritis (RA) and SLE.

Murgia et al. [16] compared SSc and HCs using NMR and GC-MS techniques. In NMR they detected aberrated metabolites that indicated aberrations in glycolysis/gluconeogenesis, energetic pathways, synthesis and degradation of ketones, amino-acid, and pyruvate metabolism. Interestingly the authors presented only 3 metabolites aberrated according to both methods (citrate, alanine, and aspartate). Those metabolites were tested to have good prognostic power and may become new biomarkers for SSc. Moreover, the researchers presented differences between major clinical subgroups of SSc. The following metabolites were increased in dcSSc: acetate, fructose, glutamate, glycerol, lysine, valine, glycerate, and glutarate. In lcSSc, these were: lactate, glutamine, sugars, and sorbitol. The authors created a differentiating metabolic model consisting of 16 metabolites and proved its diagnostic value.

One study performed amino acids and their derivatives targeted analysis Smoleńska et al. [17] searched specifically for amino-acid aberrations. The authors presented correlations of detected disturbances with the clinical presentation of SSc. The dcSSc type showed elevated sarcosine, β-alanine, MNA, and L-NAME (N-nitroarginine methyl ester) concentrations. Calcinosis was positively correlated with sarcosine, glutamate, proline, tyrosine, 3-methylhistidine, and ornithine levels. Biomarkers for arthralgia were 1-methylhistidine, ornithine (positively associated), and glutamate (negatively), whereas for ILD or PAH valine, arginine. L-NAME, glutamate, and lysine were biomarkers for telangiectasias. A negative correlation with skin hardening was detected for sarcosine, proline, histidine, ornithine, asparagine, citrulline, and phenylalanine. Moreover, they reported an increase of NO synthase inhibitor asymmetric dimethylarginine (ADMA) in SSc patients, which supports the concept of endothelium impairment as the etiology of SSc.

The last included paper [8] focused on patients with systemic lupus erythematosus (SLE), but additionally, proposed biochemical models that could differentiate SSc from SLE or HCs. According to the 25 metabolites (alanine, aminomalonic acid, arachidonic acid, arginine, aspartic acid, β-alanine, cholesterol, cysteine, cystine, glutamine, inositol-1-phosphate, lactic acid, lauric acid, malic acid, nanoic acid, oleamide, ornithine-1,5-lactam, picolinic acid, pyroglutamic acid, ribose, succinic acid, taurine, threonic acid, urea, uric acid) significantly differed in SSc vs. HCs.

### Metabolomic studies oriented to systemic sclerosis-associated pulmonary arterial hypertension (SSc-PAH)

Alotaibi et al. [18] analyzed plasma from SSc patients with and without recognized PAH and from patients with idiopathic pulmonary arterial hypertension (IPAH). They found 9 metabolites (lignoceric acid, nervonic acid, fatty acyl esters of hydroxy fatty acid, nitrooleate, 11-testosterone, 17β-estradiol, novel eicosanoid, prostaglandin F_2ɑ_, leukotriene B_4_) distinctive for SSc-PAH. Those aberrations support the thesis of dysregulated fatty acids, steroid hormones, and arachidonic acid metabolism. Furthermore, the authors tested whether those aberrations were associated with the SSc diagnosis itself, and reported that lignoceric acid and leukotriene B_4_ are in higher concentrations in SSc without pulmonary hypertension. Moreover, they presented some markers associated with SSc-PAH severity.

The next three pieces of research included in this review analyzed pulmonary arterial blood in metabolomic assessment. Deidda et al. [19] using the NMR technique proved that SSc-PAH patients can be separated from non-PAH SSc patients using metabolomic methods. They reported that SSc-PAH patients presented increased levels of acetoacetate, alanine, lactate, VLDL, and LDL, and decreased γ-aminobutyrate, arginine, betaine, choline, creatine/creatinine, glucose, glutamate/glutamine, glycine, histidine, phenylalanine, and tyrosine.

A later study [20] presented that dysregulation of kynurenine pathway correlated with PAH diagnosis, and even preceded its diagnosis. As tryptophan/kynurenine disturbance may correspond to endothelial damage, the authors additionally sought its association with Raynaud phenomenon or the presence of telangiectasias – no correlation was found.

## Discussion

This narrative review has identified 11 articles focusing on serum or plasma metabolites in SSc patients. Notably, all authors found significant differences between SSc and controls, but reported data is conflicting. Most of them prepared differentiating metabolomic models.

It is noteworthy that, besides the progress in the identification of metabolic features of systemic sclerosis data analysis algorithms are being developed. Especially the application of machine learning has been evaluated for processing metabolomic data in autoimmune diseases [21, 22]. These studies were found to provide a promising approach for precision medicine relevant to systemic sclerosis.

Seven studies searched for a correlation between clinical subtypes or organ involvement. The most studied organ complications were skin involvement, ILD, and PAH. Moreover, we found three papers, focused specifically on SSc-PAH metabolomic, which identified biomarkers associated, among others, with dysregulated steroid hormones, arachidonic acid, and other fatty acids metabolism. Noteworthy in those studies, samples were collected from pulmonary arteries. Therefore, further research on venous blood samples is necessary to interpret those results in the light of other studies. Five studies searched for ILD biomarkers in SSc. Four proposed distinctive metabolomic features, but only one metabolite was a common result in two papers (androsterone sulfate). One of the studies did not find any significant aberration in ILD patients vs. non-ILD. The results of research on other organ involvement were not consistent in the presented reports or did not reach statistical significance. This can be explained by inconsistent patient inclusion/exclusion criteria applied and relatively small patients groups recruited. Those findings additionally highlight the diversity of SSc presentations and urge the need for meticulous, standardized patient inclusion in future research. A very interesting point was made after observing the normalization of some previously noticed disturbances after treatment. This implies that the activity of SSc or its organ involvement as well as therapy status should also be assessed in each metabolomic study.

Notwithstanding, presented data outline major disturbed metabolomic pathways: energetic (glycolysis/gluconeogenesis, synthesis and degradation of ketones, fatty acid oxidation); amino acids, pyruvate, and lipid metabolism. It is not ascertained whether those aberrations are a cause or rather a consequence of the underlying pathological processes. As the spectrum of metabolites showing impaired amount is vast, in the following paragraph, we briefly present the possible implications only of chosen aberrations on the SSc pathology.

**Fig. 2:**
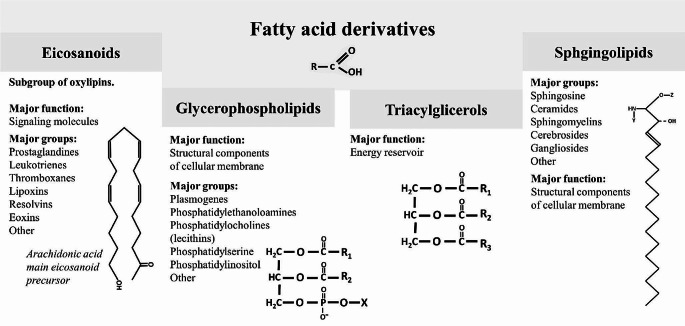
Illustrates the classification of four groups of fatty acid derivatives that hold paramount importance in human metabolism. Fatty acid derivatives substantial for human metabolism can be categorized into four subgroups presented in the scheme above. Eicosanoids belong to the broader category of lipids named oxylipins. Incapable to be stored, they are synthesized at their ultimate destination, serving as signaling molecules. Their impact ranges widely, encompassing the modulation of inflammatory processes, tissue repair, and the regulation of vasoconstriction and vasodilatation. Glycerophospholipid’s main function is building a cellular membrane. The hydrophobic tails are oriented towards the centre of the membrane, while the hydrophilic heads face its surface. Notably, glycerophospholipids serve not only as structural components but also play a role in signal transduction. Triacylglycerols function as a proficient energy reservoir. Energy is released after hydrolysis triacylglycerols into glycerol and fatty acids, through the β-oxidation process. Moreover, lipogenesis facilitates the transport of fatty acids within lipoproteins in the triacylglycerols form. Importantly, the role of triacylglycerols extends beyond dietary and energetic processes, influencing hormone balance (particularly insulin), and as a principal component of adipose tissue, they shield vital organs from potential injury. Sphingolipids constitute a diverse group, primarily known as structural components of cellular membranes. Emerging data suggests their involvement in the modulation of diverse processes including inflammation, necrosis, apoptosis, cell recognition, and autophagy. In the molecular models presented in the figure letters “R” represent the rest of the carbon chain of fatty acid; “X”, and “Z” represent the differentiating components like ethanolamine, choline, and inositol; “Y” may represent other acyl groups attached or the hydrogen atom (in sphingosine)

Lipids are a diverse group of metabolites of crucial significance as structural components of cellular membranes and energetic resources (Fig. 2). Nevertheless, their effect as signaling substances cannot be overestimated, not only for the immunological system. The elementary subgroup of lipids are fatty acids, which can be divided into three major categories: short-chain fatty acids, medium-chain fatty acids, and long-chain fatty acids. It is suggested that short-chain fatty acids may regulate T lymphocyte differentiation via modulation of histone deacetylase activity [23]. Butyrate, which has been described to be decreased in SSc patients [16, 19] is one of the short-chain fatty acids, that has a potential anti-inflammatory and antioxidant effect [24]. It can be absorbed via dietary intake but one of its major sources is gut microbiota [12]. Species known for butyrate synthesis are decreased in SSc patients, which may explain the lower level of butyrate. Though oral supplementation of butyrate may alleviate inflammation in bowel diseases or metabolic syndrome [25], the effects on SSc have not been yet investigated.

Medium-chain fatty acids present from 6 to 12 carbon atoms in the molecular chain. They are known as a quick energetic reservoir but also were proven to promote lymphocyte T differentiation (Th1 and Th17), and inhibition of Treg cells [26]. In studies included in the review, there are meager reports of aberrations of caproic acid and lauric acid. Nevertheless, later studies confirmed fatty acid aberration in SSc patients and sought correlations with specific serotypes and gut microbiome profiles [27]. Long-chain fatty acids are various metabolites that serve as an energy resource, promote immune cell activation, and proliferation, as well as stimulate the production of cytokines (both pro- and anti-inflammatory) [28]. Due to the significant diversity of this group, it is beyond the scope of this paper to discuss their role in human organism. Nevertheless, it is crucial to highlight that their significance must be considered in light of the activity of their derivatives like eicosanoids, whose aberrations have been thoroughly investigated in SSc [29]. As a result, some of them, like prostacyclin analogs, are already included in the treatment guidelines for patients with connective tissue disease (CTD) associated with PAH [30]. Noteworthy multiple disturbances in acylcarnitines were described implicating that SSc was associated with a generally disturbed process of fatty acids beta-oxidation in the mitochondria.

The other group of lipid metabolites that were found to be altered were glycerophospholipids and their derivatives. Some of them play a role in mediating the inflammatory process (like oxidated lysophospholipids), while others are responsible for the fluidity of the cellular membrane (which when disturbed may lead to impaired microcirculation) [31]. To our knowledge, no treatment targets were proposed for the aberrations of glycerophospholipids in SSc.

LPC released from LDL as a result of the action of phospholipase A2 can regulate human immunity via inducing dendritic cell maturation and promoting monocyte, phagocyte, and lymphocyte T chemotaxis [32]. There are scarce findings about their aberrations in SSc patients. Certain LPCs were detected at decreased levels, with only one exception (LPC 18:1 was found elevated) [9, 13]. There is a need for further targeted research as it may also elucidate the process of endothelial damage in this group of patients.

Lipid alterations were also present in the endocannabinoid pathway. Currently, there is growing evidence of significance for modulating fibrosis, immunity, and angiopathy [29]. Thus, promising therapeutic targets are tested on animal models [33].

Subsequently, sphingomyelins were detected in decreased levels. Moreover, this disturbance correlates with skin involvement [15]. Their significance in tissue repair and promoting fibrosis has been investigated [34], yet we lack the therapeutic targets that could be addressed for SSc treatment.

Sphingosine-1-phosphate is a lipid-signaling molecule. Its aberrations are related to disturbances in the concentration of sphingolipids, especially ceramides. Additionally, they may lead to pathological angiogenesis and increased stimulation of lymphocytes, monocytes, and fibroblasts [35].

The disturbance of amino acid metabolism in SSc has been proven by several studies, notwithstanding, the presented results differ among the authors, probably due to clinically heterogeneous patient groups included in the research. As analysis of the role of amino acids in human immunology would require a separate paper, below we discuss the implications of the most ascertained aberrations. The data presented above is consistent that SSc patients tend to present lower levels of tryptophan. In inflamed or neoplastic tissue, this essential amino acid is metabolized via three major pathways: the kynurenine, the serotonin, and the indole-3-pyruvate pathway [36]. The first one is responsible for the degradation of 95% of this amino acid, and the process is stimulated by proinflammatory agents like lipopolysaccharide, tumor necrosis factor α, and interleukin 1, and 2 [37]. The final product of this process (kynurenine) is known to stimulate the CD4 and CD8 double negative lymphocytes T and, therefore stimulate the inflammatory process [38]. Additionally, tryptophan has been suggested as a marker of endothelial damage [37]. Moreover, studies are confirming, that disturbances in the kynurenine pathway may be the marker of developing PAH in SSc patients) [20]. Those findings may provide novel avenues for treatment, especially having promising reports from research on other CTDs like SLE [39].

Finally, it is worth mentioning other modern disciplines developed lately. Genomics, transcriptomics, and proteomics have also been applied in SSc studies. Few reports on suspected “permissive genetic patterns” and meticulous analysis of SSc-proteome have been published so far. There is great potential in applying all 4 techniques (genomics, transcriptomics, metabolomics, and proteomics) simultaneously, it may lead to a thorough understanding of the disease pathology. To our knowledge, no such research was presented at the time of writing this review. Nevertheless, a narrative review [40] meticulously covers this problem leading to the conclusion that SSc patients are a highly heterogeneous group and more integrated studies are necessary to improve the personalized approach.

## Conclusion

In conclusion, metabolomic aberrations are present in systemic sclerosis patients and may become promising biomarkers. Nevertheless, further study with restrictive inclusion criteria and comprehensive organ involvement assessment is necessary to ascertain the previously reported findings and to enable its implementation into clinical practice.

.
